# Dynamics of Fecal Microbiota with and without Invasive Cervical Cancer and Its Application in Early Diagnosis

**DOI:** 10.3390/cancers12123800

**Published:** 2020-12-16

**Authors:** Gi-Ung Kang, Da-Ryung Jung, Yoon Hee Lee, Se Young Jeon, Hyung Soo Han, Gun Oh Chong, Jae-Ho Shin

**Affiliations:** 1 Department of Applied Biosciences, Kyungpook National University, Daegu 41566, Korea; gukang@knu.ac.kr; 2 Department of Biomedical Convergence Science & Technology, Kyungpook National University, Daegu 41566, Korea; amugae1210@knu.ac.kr; 3 Department of Obstetrics and Gynecology, School of Medicine, Kyungpook National University, Daegu 41404, Korea; mylyh3@naver.com; 4 Department of Obstetrics and Gynecology, Kyungpook National University Chilgok Hospital, Daegu 41404, Korea; tpqkf0927@naver.com; 5 Clinical Omics Research Center, School of Medicine, Kyungpook National University, Daegu 41940, Korea; hshan@knu.ac.kr; 6 Department of Physiology, School of Medicine, Kyungpook National University, Daegu 41405, Korea

**Keywords:** machine learning, gut microbiome, vaginal microbiome, prediction

## Abstract

**Simple Summary:**

The fecal microbiome has been suggested to be linked to invasive cervical cancer (ICC). Considering that ICC is common in women, it is important to identify bacterial signatures from fecal microbiota that contribute in classifying cervical cancer. Although previous studies have suggested possible biomarkers based on fecal microbiota, limited information exists in terms of the diagnostic ability using gut microbiota-derived signatures for detecting early ICC. The purpose of this study was to investigate the potential association between early ICC and fecal microbiota and to examine whether fecal microbiota-derived markers can be utilized as a non-invasive tool to diagnose early ICC using machine learning (ML) techniques. Further studies to incorporate quantitative and qualitative characterization of identified individual bacterial genus and validate our model in larger cohorts are imperative in terms of causality for the association between cervical cancer and microbes.

**Abstract:**

The fecal microbiota is being increasingly implicated in the diagnosis of various diseases. However, evidence on changes in the fecal microbiota in invasive cervical cancer (ICC) remains scarce. Here, we aimed to investigate the fecal microbiota of our cohorts, develop a diagnostic model for predicting early ICC, and identify potential fecal microbiota-derived biomarkers using amplicon sequencing data. We obtained fecal samples from 29 healthy women (HC) and 17 women with clinically confirmed early ICC (CAN). Although Shannon’s diversity index was not reached at statistical significance, the Chao1 and Observed operational taxonomic units (OTUs) in fecal microbiota was significantly different between CAN and HC group. Furthermore, there were significant differences in the taxonomic profiles between HC and CAN; *Prevotella* was significantly more abundant in the CAN group and *Clostridium* in the HC group. Linear discriminant analysis effect size (LEfSe) analysis was applied to validate the taxonomic differences at the genus level. Furthermore, we identified a set of seven bacterial genera that were used to construct a machine learning (ML)-based classifier model to distinguish CAN from patients with HC. The model had high diagnostic utility (area under the curve [AUC] = 0.913) for predicting early ICC. Our study provides an initial step toward exploring the fecal microbiota and helps clinicians diagnose.

## 1. Introduction

Invasive cervical cancer (ICC) is one of the common health problem for women worldwide, affecting approximately 500,000 women every year [1]. Human papillomavirus (HPV) is the major cause of 95–100% of cases of ICC [2]. Although HPV infection is the primary cause, it does not determine the development of cervical cancer. Most HPV infections are cleared, and only a small proportion of infected women develop cervical intraepithelial neoplasia or ICC. Thus, understanding tumorigenesis is still insufficient. Martin et al. [3] demonstrated that complex host variations are significant in the development of carcinogenesis.

The intestinal microbiota co-exist with their hosts symbiotically and play an essential role in host health and disease. Studies have shown that fecal microbes orchestrate not only the host’s metabolism but also their immune response. Several attempts have been made to theorize the association between intestinal microbiota and ICC [4,5,6,7]. Sims et al. [8] reported differences in fecal microbial composition between patients with ICC and healthy controls, indicating that their gut microbiota reflect etiologic or clinical differences by age. Wang et al. [9] demonstrated Proteobacteria, *Parabacteroides*, *Escherichia*–*Shigella*, and *Roseburia* as possible biomarkers for ICC diagnosis.

Although these studies demonstrated the potential association between fecal microbiota and ICC, identifying biomarkers to predict ICC remains a challenge. The number of bacterial types found within the fecal microbiota is complex [10] and the number of possible correlations between these bacteria are more complex. Moreover, the relationship between the fecal microbiota and ICC might be obscured by variations in data [11]. Not only this, the fact that different bacterial compositions may provide similar functionality is also one of the difficulties [12]. These problems are analogous to the challenges faced by the geneticists [13], where there are a number of genetic interactions that can be associated with cervical cancer, making it difficult to determine the causative agents [14].

Given the importance of the ICC, and the potential relationship between fecal microenvironment and cervical health, there exists a powerful rationale or the development of a prediction model using fecal microbiota-based biomarkers that might be used to predict early ICC. We investigated the fecal microbiota of a well-characterized cohort of participants with biopsy-proven patients. Furthermore, we applied machine learning (ML) algorithms to discover possible interactions associated with early ICC and identify potential microbial markers. In particular, we aimed to develop a prediction model for early ICC diagnosis in the form of a classifier.

## 2. Results

### 2.1. Participants’ Characteristics

This study enrolled 46 participants in total, 29 healthy women (HC) and 17 patients with invasive cervical cancer (CAN). The HC group included healthy volunteers, aged 20–54 years, without a symptomatic history of the reproductive tract for the last 10 years. A total of 17 of patients with early stage-cervical cancer, aged 29–64 years, were recruited before the loop electrosurgical excision procedure in our hospital between January and August 2020. Additional patients’ characteristics are presented in Table 1.

### 2.2. Dynamics of Fecal Microbiota

**Figure 1:**
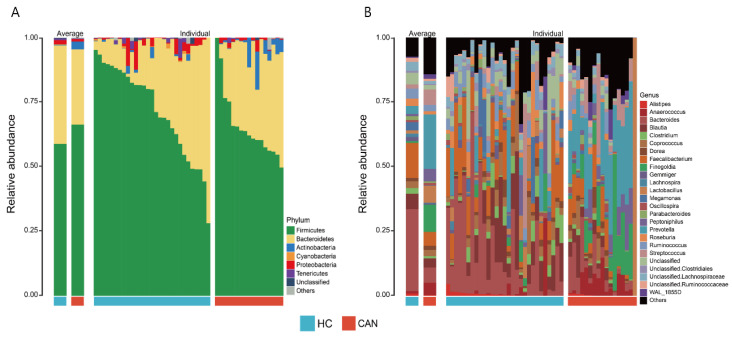
Relative abundance of fecal microbiota composition at the phylum (**A**) and genus (**B**) levels by health statuses (HC vs. CAN). The left panel of each figure represents the average relative abundance. The right panel displays the individual relative abundance of each sample. The top seven phyla and top 25 genera are presented.

**Figure 2:**
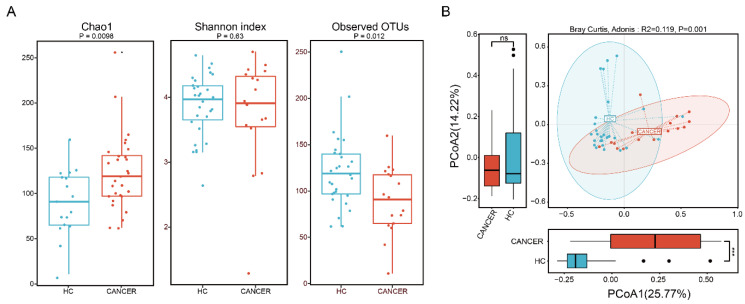
The α- and β-diversity of fecal microbiota by group (HC vs. CAN). (**A**) Significant differences in Chao1 and observed OTUs were observed between each group (*p* = 0.0098 and *p* = 0.012). No significant difference was observed between HC and CAN regarding Shannon’s index. (**B**) Principal coordinates analysis (genus level) using the Bray–Curtis dissimilarity, displaying the dissimilarities of fecal microbiota between each group.

A total of 46 fecal microbial samples from 46 participants were examined. A median of 14,315 (ranging from 5953 to 125,338) high-quality sequences were obtained after performing quality control using DADA2 software. Figure 1 shows taxonomic composition at the phylum (Figure 1A) and genus (Figure 1B) levels, stratified by health status (HC vs. CAN). The fecal microbial composition was mainly dominated by Firmicutes (60.86%), Bacteroidetes (35.73%), and Actinobacteria (1.02%) at the phylum level and *Bacteroides* (24.74%), *Faecalibacterium* (11.33%), *Prevotella* (7.95%), and *Blautia* (5.31%) at the genus level. To evaluate differences in intestinal microbial α-diversity (i.e., Chao1, Shannon’s index, and observed OTUs), we compared the diversity and richness indices of HC and CAN (Figure 2A). A comparison of the Chao1 presented a significant reduction in HC (*p* = 0.0098). Opposite trend was shown in observed OTUs (*p* = 0.012). However, no statistical significance in Shannon’s diversity was observed (*p* = 0.63). To evaluate the extent of dissimilarity in fecal microbial composition, β-diversity was computed based on the Bray–Curtis distance. The principal coordinates analysis (PCoA) plot was used for presenting the microbial composition of each group, and ADONIS analysis was performed to compare the dispersion among health statuses. Upon analyzing β-diversity at the genus level, we observed significant differences (*p* = 0.001) in clustering of each group in the PCoA plot (Figure 2B). Upon close examination of the PCoA1 and PCoA2 axis of our samples, we observed significant differences in PCoA1 (*p* < 0.001 by the Wilcoxon test). However, no significant difference was observed in PCoA2.

### 2.3. Comparative Analysis of the Fecal Microbial Taxa between HC and CAN

**Figure 3:**
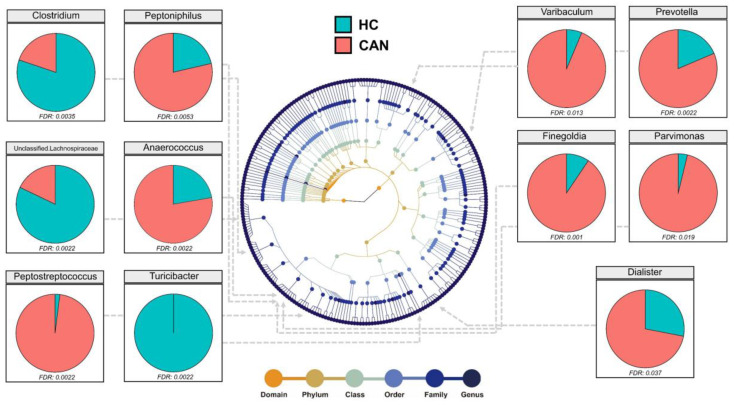
Differential abundance analysis of fecal microbiota at the genus level. Only statistically significant genera were displayed (Wilcoxon rank sum test, False discovery rate (FDR) < 0.05). Different colored points in the phylogeny represent taxonomic levels.

**Figure 4:**
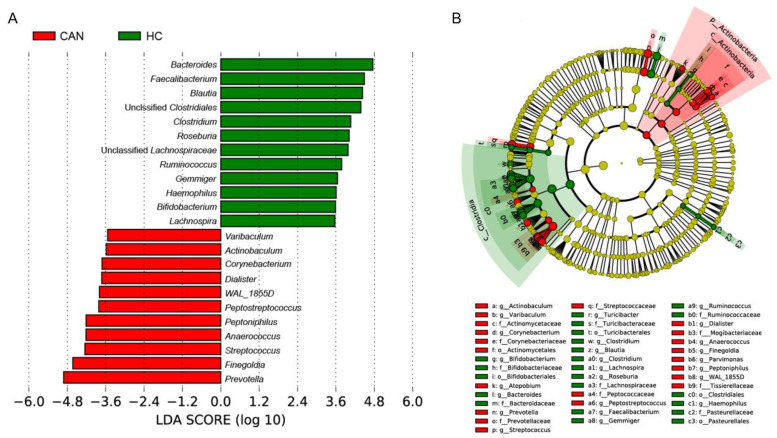
Linear discriminant analysis (LDA) effect size (LEfSe) analysis for fecal microbiota abundance based on clinical features (HC vs. CAN). (**A**) Shows the representative genera for HC and CAN. (**B**) Cladogram of taxa associated with HC and CAN.

Assigned sequences were used to evaluate differences in taxonomic abundances between CAN and HC at various taxonomic levels. We observed significant changes in fecal microbiota structures between HC and CAN. At the phylum level, the abundance of Bacteroidetes, Firmicutes, and Proteobacteria did not reach statistical significance (data not shown). At the family level, the abundance of *Lachnospiraceae* (*p* = 0.001) and *Turicibacteraceae* (*p* = 0.0017) in HC was significantly higher, whereas the abundance of *Tissierellaceae* (*p* = 0.001), *Prevotellaceae* (*p* = 0.001), and *Actinomycetaceae* (*p* = 0.0023) was higher in CAN (Figure S1). At the genus level, *Prevotella* (*p* = 0.0022) was notably more abundant in CAN. While Unclassified *Lachnospiraceae* (*p* = 0.0022) and *Clostridium* (*p* = 0.0035) were more abundant in HC. Other genera, namely *Finegoldia*, *Anaerococcus*, *Peptostreptococcus*, *Peptoniphilus*, *Varibaculum*, *Parvimonas*, and *Dialister* were enriched in the CAN group, and the difference was statistically significant (Figure 3). Linear discriminant analysis effect size (LEfSe) analysis was performed to compare the most differently abundant taxa of each group (Figure 4). Linear discriminant analysis (LDA) scores that exceeded 3.5 were obtained as the representative genus in each group, revealing relatively consistent results with that of abundance analysis (Figure 3). Both the LDA graph (Figure 4A) and the cladogram (Figure 4B) showed that 11 genera (*Varibaculum*, *Actinobaculum*, *Corynebacterium*, *Dialister,* WAL_1855D, *Peptostreptococcus*, *Peptoniphilus*, *Anaerococcus*, *Streptococcus*, *Finegoldia*, and *Prevotella*) were more abundant in the CAN group, whereas 12 genera (*Bacteroides*, *Faecalibacterium*, *Blautia*, Unclassified Clostridiales, *Clostridium*, *Roseburia*, Unclassified Lachnospiraceae, *Ruminococcus*, *Gemmiger*, *Haemophilus*, *Bifidobacterium*, and *Lachnospira*) were more abundant in the HC cluster.

### 2.4. Ecological Network and Correlation Analysis

**Figure 5:**
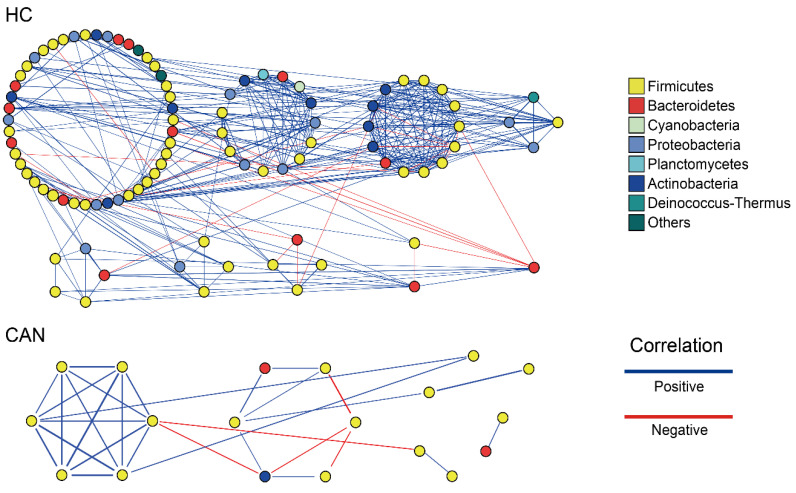
Network analysis of fecal microbiota. The symbiotic pattern in the HC and CAN groups when rho >0.5 and *p* < 0.05 at the genus level. (Blue and red lines represent positive and negative correlation, respectively. All clusters are colored by phylum).

The symbiotic network of each group was built based on Spearman’s correlation coefficient for genera with rho values (correlation coefficient) more than 0.5 and *p* < 0.05 (Figure 5). One module included a group of genera that are connected between themselves, but had much fewer connections with genera outside the group. As shown in Figure 5, in the HC group, the symbiotic network consisted of nine modules with 49 nodes (genera) and 172 edges; five of the nine modules with ≥5 nodes were obtained from network analysis, and most relationships were positive. By contrast, in the CAN group, only six modules were obtained from network analysis, with 12 nodes and 23 edges. Only two modules had ≥5 nodes; overall, they also showed a positive relationship. We then investigated the potential correlation between disease features and fecal microbiota composition to explore if it provides the heterogeneity of the microbial community (Figure S2). Within the HC group, Firmicutes and Bacteroidetes positively correlated with each other (rho = 0.83, *p* < 0.001). By contrast, in the CAN group, Bacteroidetes and Actinobacteria showed a slightly positive correlation (rho = 0.55, *p* < 0.05). Based on their correlation pattern, we further investigated the Firmicutes to Bacteroidetes ratio (F/B ratio). Case control studies [15,16,17,18,19] have demonstrated that higher ratios are observed in patients with diseases such as hypertension, chronic fatigue syndrome, and autism. However, this trend was not seen in our cohort (*p* = 0.182).

### 2.5. Predictive Model Based on Fecal Microbiota

**Figure 6:**
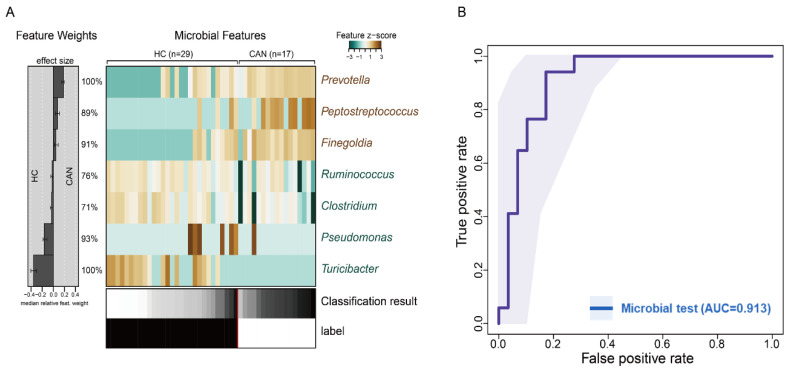
Machine learning analysis. (**A**) Relative abundance of seven fecal-derived genera potentially associated with ICC is presented as a heatmap. The effect size of each marker genera for the classification is displayed to the left. (**B**) Mean test prediction accuracy measured by the area under the ROC curve (AUC).

In order to determine whether differences in fecal microbial composition can be considered potential biomarkers for distinguishing CAN from HC, we applied microbiota classification based on L1-LASSO regression to our sequencing data (Figure 6A). Our model selected seven genera as the most important features (*Prevotella*, *Peptostreptococcus*, *Finegolida*, *Ruminococcus*, *Clostridium*, *Pseudomonas*, and *Turibacter*). *Prevotella* and *Turibacter* were the top predictors in our model. Area under curve (AUC) of ROC curve was computed to evaluate the predictive ability. As shown in Figure 6B, the discriminant model based on the seven genera effectively distinguishes CAN from HC (mean AUC = 0.913). From the seven genera selected, two were >3-fold abundant in HC compared to that in CAN, whereas three were >3-fold abundant in CAN (Table 2). Microbial signatures were further validated by applying random forest (RF) to the original dataset and checking for overlapped features. The trained RF selected 21 genera as the most important features, five of which overlapped with the genera selected as features in our prediction models and LEfSe analysis (Figure S3). Furthermore, the RF model showed considerably high prediction accuracy (AUC = 0.91 and 0.88 in the training and test sets, respectively, Figure S4). Thus, ML-based fecal microbiota could distinguish between the CAN and HC groups among our cohort, indicating that intestinal microbiota can be used as potential biomarkers to predict early-stage of ICC.

## 3. Discussion

ICC is known as a multifactorial disease that is affected by various genetic and environmental factors. Although HPV infection is critical for the incidence of cervical cancer [20], the impact of other potential factors on early stage of ICC has not been actively studied. In this study, we aimed to investigate the fecal microbiota of women with and without ICC and build a diagnostic model based on ML algorithm. We found that the dynamics of the fecal microbiota differed between healthy women and patients with early ICC. Significant differences in α- and β-diversity between patients with ICC and cancer-free controls were observed, demonstrating compositional differences in the fecal microbial community. Furthermore, we developed a ML-based prediction model to detect the presence of early ICC using the relative abundance of specific bacterial genera. Preliminary results of this study revealed that ML-based ROC analysis can predict and detect early ICC. Additionally, we demonstrated the diagnostic accuracy of fecal microbiota-based biomarkers to predict this disease. Most of the seven bacterial genera used for building this model were directly or indirectly linked to human health. Furthermore, our model had considerably high accuracy in detecting early stage of ICC (AUC = 0.913).

Recently, Wang et al. [9] reported a strong association between gut microbiome and cervical cancer by comparing the gut microbial composition between five healthy controls and eight patients with cervical cancer. The authors demonstrated that in patients with cervical cancer, α-diversity had an increasing trend, and a clear separation was found between each group for β-diversity. Moreover, they identified several gut microbial compositions at various taxonomic levels. For example, Proteobacteria was significantly higher in abundance in the cancer group, and *Escherichia*–*Shigella*, *Roseburia*, *Pseudomonas*, *Lachnoclostridium*, Lachnospiraceae_UCG-004, *Dorea*, Unidentified Lachnospiraceae, *Fusicatenibacter*, Lachnospiraceae_UCG-010, *Yersinia*, and *Succinivibrio* had a significantly higher abundance at the genus level. However, our analysis of fecal microbiota in healthy women vs. patients with ICC is in contrast with that of Wang et al. We observed significantly lower observed OTUs in patients with cervical cancer. Although there was no statistical significance, Shannon’s index was lower. Furthermore, our analysis on compositional differences, in terms of the fecal microbial community according to health statuses, showed dissimilarities in the abundance of specific genera in patients with cervical cancer. Demographic characterization of each cohort and different bioinformatic methods for identifying bacterial composition may cause dissimilarities [21,22,23]. Bacterial identification performed by the previous study was amplified using the 16S rRNA gene with primers targeting the V4 region [9]. However, primer pairs targeting the V4-V5 region were used in our study, which might contribute to the differential abundance of bacterial genera.

Emerging studies have suggested the potential links between the increased abundance of *Prevotella* and several disorders, such as bacterial vaginosis, metabolic disorders, low-grade systemic inflammation, and periodontitis [24,25,26,27,28]. Larsen et al. [29] demonstrated the association between the abundance of *Prevotella* and mucosal inflammation mediated by T helper type 17 (Th17). Other studies have also supported the role of *Prevotella.* Gosmann et al. [30] revealed that in the vaginal tract, *Prevotella* contributes to the activation of a Th17 immune response. Li et al. showed a causal role of *Prevotella*-enriched gut enterotype, suggesting its contribution to a specific disease [31]. A study in mice conducted by Elinav et al. [32] revealed a potential role of *Prevotella* in the fecal microbial environment by promoting dextran sulfate sodium (DSS)-induced colitis. In the patient group of our study, *Ruminococcus* and *Clostiridium*, known as butyrate-producing bacteria, are decreased. As a major nutrient of the intestinal tract, butyrate plays important roles in controlling inflammation and preventing leaky gut and regulates intestinal autophagy and energy metabolism in the human colon [33,34,35]. Thus, a reduction of butyrate-producing bacteria may affect general intestinal health, thereby affecting vaginal health. Taken together, *Prevotella*, *Ruminococcus*, and *Clostridium* may be linked to early ICC risk.

ML techniques have been used to assess the association between the microbiome and numerous disease status [36,37,38]. Prominent studies have established the concept of fecal microbiota as non-invasive diagnostic tools for certain diseases or cancers, including hepatocellular carcinoma (CRC), nonalcoholic fatty liver disease (NAFLD), and type 2 diabetes (T2D) [39,40,41,42]. In this study, we demonstrated characteristic differences in the fecal microbiota of our cohort, identified specific seven biomarkers, constructed a prediction model, and validated its diagnostic accuracy. Thus, fecal microbiota-derived microbial markers may become potential tools for the diagnosis of early ICC. Further studies to validate fecal microbiota-derived biomarkers in larger cohorts from various ethnic populations and countries are needed to promote the accuracy and stability for early ICC diagnosis.

We acknowledge the limitations associated with our study, which include the following: (1) Our findings only provide preliminary potential of an association between fecal microbiota and early ICC compared to cancer-free controls. Therefore, we could not address whether changes in the fecal microbial community are driven by tumor development or tumor formation in terms of causal relationship, and additional studies are essential to assess how bacterial genera play a role in cervical health, affecting ICC. (2) Our study was based on 16S rRNA gene sequencing, limiting identification of bacterial species, and evaluating the role of the fecal microbiota alone. (3) Although our data support the hypothesis that *Prevotella* is associated with ICC, it does not support causality. (4) Age is a confounding variable for the incidence of ICC. However, changes in the vaginal microbiome with respect to age were not evaluated. Further studies need to validate the results of this study according to age. (5) It is well known that HPV infection affects incidence and progression of ICC. However, the results of this study did not evaluate potential association between HPV infection and alterations in fecal microbiota. Furthermore, since gut microbiota might be affected by diet and lifestyle of the individual, further studies are needed to assess how those environmental factors are correlated to fecal microbiota of ICC patients. Nonetheless, key strengths of this study include a prospective prediction model, which successfully predicted cervical cancer with a high AUC score. Moreover, we identified seven bacterial genera that are differentially abundant between normal women and patients with cancer from a well-characterized cohort based on fecal microbial composition.

## 4. Materials and Methods

### 4.1. Study Population

We obtained ethical approval from the Institutional Review Board of Kyungpook National University Chilgok Hospital (KNUMC 2015-10-033, 16-11-2015), the Armed Forces Medical Research Ethics Review Committee (AFMC-17-IRB-092, 17-11-2017), and Kyungpook National University (KNU 2017-84, 24-08-2017), Republic of Korea. Every participant provided written informed consent according to the Declaration of Helsinki. In the CAN group, patients were clinically staged according to the 2009 International Federation of Gynecologic Obstetrics (FIGO) staging system [43]. Patients with a history of preoperative chemotherapy, radiotherapy, or administration of antibiotics were excluded. Fecal samples were collected using Transwab tubes (Sigma, Dorset, UK) from healthy women and patients with early ICC. All collected fecal swabs were sent to the laboratory, and stored at −80 °C until further processing.

### 4.2. HPV-Assay and HPV Genotyping

HPV genotyping was conducted using cervico-vaginal swabs and the Anyplex II HPV 28 assay kit (Seegene, Guri, Korea) was used, following the instructions of the manufacturer. In brief, 5 μL DNA was used in each of 2 20-μL reactions with primer set A or B. In this assay, HPV-specific dual priming oligonucleotides were used for multiplex real-time PCR. A total of 28 HPV types were tested to detect 18 high-risk types (HPV 16, 18, 26, 31, 33, 35, 39, 45, 51, 52, 56, 58, 59, 66, 68, 69, 73, and 82) and eight low-risk types (HPV 6, 11, 40, 42, 44, 53, 54, and 70).

### 4.3. DNA Extraction and 16S rRNA Gene Sequencing

Total fecal DNA was extracted from each sample using the QIAamp PowerFecal Pro DNA Kit (Qiagen, Hilden, Germany), following the protocol provided by manufacturer. Bacterial DNA concentration was measured using a Qubit^®^ 2.0 Fluorometer (Life Technologies, Carlsbad, CA, USA) and the quality of extracted DNA was assessed by electrophoresis. For amplicon sequencing, DNA isolated from each sample was amplified with universal primer pairs targeting the V4–V5 regions of bacterial 16S rRNA genes, 515F (5′-barcode-GTGCCAGCMGCCGCGGTAA-3′), and 907R (5′-barcode-CCGYCAATTCMTTTRAGTTT-3′). PCR was performed according to the conditions as previously described [44]. Briefly, 95 °C for 5 min; 30 cycles of 95 °C for 30 s, 57 °C for 30 s, 72 °C for 30 s; and 72 °C for 5 min and held at 4 °C. The PCR products were purified using the QIAquick gel extraction kit (QIAGEN, Germany). Sequencing libraries were pooled in equal concentrations. To confirm the ideal concentration needed for amplicon sequencing, the Agilent 2100 Bioanalyzer (Agilent Technologies, Santa Clara, CA, USA) was used. Purified and amplified libraries were sequenced on the Ion Torrent PGM for 1250 flows with the Ion PGM™ Hi Q Sequencing Kit (Thermo Fisher Scientific, Waltham, MA, USA), according to the manufacturer’s protocol.

### 4.4. Bioinformatic Analysis

For amplicon sequencing analysis, the generated raw single-end reads were acquired from the Ion Torrent Software Suite as the FASTQ format and processed using the Quantitative Insights Into Microbial Ecology 2 (QIIME2) v. 2020.8 software [45]. Sequences were then processed through quality filtering, trimming, dereplicating, and denoising using DADA2 for obtaining amplicon sequence variants (ASV) [46]. Briefly, the quality filtering was conducted according to their mean frequency of 18,768 and the Q score (sequencing quality) that is less than 30 was trimmed and denoised. The ASVs at an abundance that is less than 0.1% of the mean sample depth were filtered prior to further analysis. Non-bacterial, mitochondrial, and chloroplast sequences were removed. Representative sequences were then assigned for taxonomic identification using a custom trained naïve Bayes ML classifier, trained for differentiating taxa present with the 99% cutoff value using against the Greengenes 13_8 database. Sequences were rarefied at a minimum sequencing depth of 5953 reads. All samples in the OTU table were subsampled to equal depths prior to further analysis.

### 4.5. Statistical Analysis

General statistical analyses and visualization of our sequencing results were carried out using RStudio 1.0.153 (https://www.rstudio.com/) and Calypso web application [47]. Alpha diversity indexes within the samples were evaluated using an ANOVA test to measure statistical significance among two different groups (HC vs. CAN). PCoA was performed to analyze and visualize patterns of β-diversity based on the Bray–Curtis dissimilarity. Two-dimensional PCoA analysis was conducted using R with the vegan, reshape, and ggplot2 packages [48,49,50]. Because each axis in PCoA has a unique value that represents the degree of variation in that axis, we represented statistical difference of each axis between groups. Phylogenies were manipulated using GraPhlAn [51]. The difference between each group at the various taxonomic level was calculated using Wilcoxon rank-sum test. To further control error, the false discovery rate (FDR) was applied and genera with a FDR of <0.05 were considered differentially abundant and visualized using a pie chart. In addition, the symbiotic network relationship between each group’s microbiota was computed and displayed using Cytoscape (v3.7.2) according to Spearman’s correlation coefficient [52]. To discover biomarkers or genomic features that characterize the differences between different biological conditions, LDA effect size (LEfSe) was conducted based on the Huttenhower Galaxy web application (http://huttenhower.sph.harvard.edu/galaxy/). For this analysis, the Kruskal–Wallis test was used to detect features with significant differential abundance among classes. Abundance differences of bacterial genus between HC vs. CAN clusters were identified using the LEfSe approach. Then, LDA was performed to evaluate the effect size of each feature (*p* < 0.05 and LDA score >3.5). The correlations between the phyla within each group were estimated using Spearman’s correlation coefficient and plotted using the Corrplot and PerformanceAnalytics packages [53,54]. To test the association between the composition of fecal microbiota and cervical cancer, the relative abundance was log-transformed and normalized to z-scores for model construction. In a five-fold cross-validation setup with 10 iterations, an L1 normalized (LASSO) logistic regression model [55] was applied to the training set and then evaluated on the test set within every cross validated fold. Every machine learning step including data preprocessing, model construction, feature selection, and evaluation of final model was performed using the SIAMCAT package [56]. To validate the bacterial signature for distinguishing between HC and CAN, we constructed an additional ML model based on RF to build a classifier from the same sample set with the randomForest package [57]. Briefly, the sample set was randomly split into two sets with same proportion of each group (29 samples for training and 17 for test sets) as described by Chakravarthy et al. [58]. The model was constructed using genera abundances with default parameters. Feature selection was performed by the iterative feature elimination step to optimize this model and the final model was built based on the selected features using caret package [59]. AUC of ROC curve was calculated to measure the accuracy of the classifier. A Venn diagram was drawn to present overlapped potential biomarkers between MLs and LEfSe analysis using VennDiagram package [60].

## 5. Conclusions

In conclusion, we suggest an association between fecal microbiota and ICC. Furthermore, ML-based on fecal microbiota can aid women for the prevention of cervical cancer in terms of diagnosis. Although challenges remain in advancing the knowledge of fecal microbiota into the clinic for early ICC diagnosis, our findings provide an initial step toward exploring fecal microbiota for clinician’s decision-making and monitoring early ICC.

## Figures and Tables

**Table 1:** Patients’ characteristics.

Variables	HC (*n* = 29)	CAN (*n* = 17)	*p* Value
Age (years)	36.1 ± 9.9	43.9 ± 9.9	0.02
Menopause (*n*, %)	2 (6.9)	6 (35.3)	0.04
Marriage (*n*, %)	5 (17.2)	16 (94.1)	<0.0001
Smoker (*n*, %)	10 (34.5)	5 (29.4)	0.005
Contraceptive use (*n*, %)	8 (27.6)	2 (11.8)	0.282
HPV positive (*n*, %)	0 (0)	17 (100)	<0.0001
High-risk HPV positive (*n*, %)	0 (0)	13 (76.5)	<0.0001
Cervical cancer severity	NA		NA
FIGO * stage (*n*, %)			
IA1		14 (82.3)	
1A2		1 (5.9)	
IB1		2 (11.8)	

* FIGO = International Federation of Gynecologic Obstetrics; NA = Not available.

**Table 2:** Important genera selected by our machine learning model.

Genus	HC (Mean)	CAN (Mean)	Foldchange	*p* Value
*Prevotella*	13.98	51.01	3.65	0.0022
*Peptostreptococcus*	0.62	9.78	15.67	0.0022
*Finegoldia*	6.07	32.85	5.41	0.001
*Ruminococcus*	27.59	15.3	1.8	0.058
*Clostridium*	20.99	9.48	2.22	0.0035
*Pseudomonas*	1.96	0.64	3.04	0.49
*Turicibacter*	3.01	0	21,333.84	0.0022

## Supplementary Materials

The following are available online at https://www.mdpi.com/2072-6694/12/12/3800/s1, Figure S1: Box-plots showing difference of bacterial abundance at the family level. All samples in each group were visualized by colored dots. The lines in the boxes represented median of values. *p* values were computed to compare their statistical significance by Wilcoxon test and adjusted using false discovery rate (FDR) procedure. Figure S2: Correlation matrix of fecal microbiota composition by phylum within each group. The distributions of each phyla are displayed on the diagonal in the form of histogram based on kernel density estimation. The bottom of the diagonal represents scatter plots with fitted line. Positive and negative correlations are shown in positive and negative numbers, respectively. (A) HC, (B) CAN. * *p* < 0.05; ** *p* < 0.1; *** *p* < 0.005. Figure S3: Venn diagram depicting the overlap of potential biomarkers selected by each of feature selection methods. Five genera were overlapped with features selected using the LASSO regression-based prediction model. Figure S4: ROC curves presenting predictive accuracy based on random forest analysis. (A) ROC curve of training set (B) ROC curve of test set. Each set was evaluated 300 times and mean AUC are presented. Grey lines and box plots present each of 300 runs. The black line represents median of those values.

## References

[1] Waggoner S.E. (2003). Cervical cancer. Lancet.

[2] Okuma K., Yamashita H., Yokoyama T., Nakagawa K., Kawana K. (2016). Undetected human papillomavirus DNA and uterine cervical carcinoma. Strahlenther. Und Onkol..

[3] Martin C., Kehoe L., Spillane C., O’Leary J. (2007). Gene discovery in cervical cancer: Towards diagnostic and therapeutic biomarkers (vol 11, pg 277, 2007). Mol. Diagn. Ther..

[4] Fan Y., Pedersen O. (2021). Gut microbiota in human metabolic health and disease. Nat. Rev. Microbiol..

[5] Martin A.M., Sun E.W., Rogers G.B., Keating D.J. (2019). The influence of the gut microbiome on host metabolism through the regulation of gut hormone release. Front. Physiol..

[6] Wu H.-J., Wu E. (2012). The role of gut microbiota in immune homeostasis and autoimmunity. Gut Microbes.

[7] Zheng D., Liwinski T., Elinav E. (2020). Interaction between microbiota and immunity in health and disease. Cell Res..

[8] Sims T.T., Colbert L.E., Zheng J., Medrano A.Y.D., Hoffman K.L., Ramondetta L., Jazaeri A., Jhingran A., Schmeler K.M., Daniel C.R. (2019). Gut microbial diversity and genus-level differences identified in cervical cancer patients versus healthy controls. Gynecol. Oncol..

[9] Wang Z., Wang Q., Zhao J., Gong L., Zhang Y., Wang X., Yuan Z. (2019). Altered diversity and composition of the gut microbiome in patients with cervical cancer. AMB Express.

[10] Donaldson G.P., Lee S.M., Mazmanian S.K. (2016). Gut biogeography of the bacterial microbiota. Nat. Rev. Microbiol..

[11] Bundgaard-Nielsen C., Hagstrøm S., Sørensen S. (2018). Interpersonal variations in gut microbiota profiles supersedes the effects of differing fecal storage conditions. Sci. Rep..

[12] Mobeen F., Sharma V., Prakash T. (2020). Comparative gut microbiome analysis of the Prakriti and Sasang systems reveals functional level similarities in constitutionally similar classes. 3 Biotech.

[13] Beck D., Foster J.A. (2014). Machine learning techniques accurately classify microbial communities by bacterial vaginosis characteristics. PLoS ONE.

[14] Martinez-Nava G.A., Fernandez-Nino J.A., Madrid-Marina V., Torres-Poveda K. (2016). Cervical cancer genetic susceptibility: A systematic review and meta-analyses of recent evidence. PLoS ONE.

[15] Heshiki Y., Vazquez-Uribe R., Li J., Ni Y., Quainoo S., Imamovic L., Li J., Sørensen M., Chow B.K., Weiss G.J. (2020). Predictable modulation of cancer treatment outcomes by the gut microbiota. Microbiome.

[16] Mariat D., Firmesse O., Levenez F., Guimarăes V., Sokol H., Doré J., Corthier G., Furet J. (2009). The Firmicutes/Bacteroidetes ratio of the human microbiota changes with age. Bmc Microbiol..

[17] Woting A., Blaut M. (2016). The intestinal microbiota in metabolic disease. Nutrients.

[18] Yang T., Santisteban M.M., Rodriguez V., Li E., Ahmari N., Carvajal J.M., Zadeh M., Gong M., Qi Y., Zubcevic J. (2015). Gut dysbiosis is linked to hypertension. Hypertension.

[19] Zhang M., Ma W., Zhang J., He Y., Wang J. (2018). Analysis of gut microbiota profiles and microbe-disease associations in children with autism spectrum disorders in China. Sci. Rep..

[20] Reid R., Stanhope C.R., Herschman B.R., Booth E., Phibbs G.D., Smith J.P. (1982). Genital warts and cervical cancer. I. Evidence of an association between subclinical papillomavirus infection and cervical malignancy. Cancer.

[21] Schloss P.D., Jenior M.L., Koumpouras C.C., Westcott S.L., Highlander S.K. (2016). Sequencing 16S rRNA gene fragments using the PacBio SMRT DNA sequencing system. PeerJ.

[22] Bukin Y.S., Galachyants Y.P., Morozov I., Bukin S., Zakharenko A., Zemskaya T. (2019). The effect of 16S rRNA region choice on bacterial community metabarcoding results. Sci. Data.

[23] Johnson J.S., Spakowicz D.J., Hong B.-Y., Petersen L.M., Demkowicz P., Chen L., Leopold S.R., Hanson B.M., Agresta H.O., Gerstein M. (2019). Evaluation of 16S rRNA gene sequencing for species and strain-level microbiome analysis. Nat. Commun..

[24] Anahtar M.N., Byrne E.H., Doherty K.E., Bowman B.A., Yamamoto H.S., Soumillon M., Padavattan N., Ismail N., Moodley A., Sabatini M.E. (2015). Cervicovaginal bacteria are a major modulator of host inflammatory responses in the female genital tract. Immunity.

[25] Pedersen H.K., Gudmundsdottir V., Nielsen H.B., Hyotylainen T., Nielsen T., Jensen B.A., Forslund K., Hildebrand F., Prifti E., Falony G. (2016). Human gut microbes impact host serum metabolome and insulin sensitivity. Nature.

[26] Goh C.E., Kopp J., Papapanou P.N., Molitor J.A., Demmer R.T. (2016). Association between serum antibodies to periodontal bacteria and rheumatoid factor in the Third National Health and Nutrition Examination Survey. Arthritis Rheumatol..

[27] Dahlén G. (1993). Black-pigmented gram-negative anaerobes in periodontitis. FEMS Immunol. Med Microbiol..

[28] Berezow A.B., Darveau R.P. (2011). Microbial shift and periodontitis. Periodontology 2000.

[29] Larsen J.M. (2017). The immune response to Prevotella bacteria in chronic inflammatory disease. Immunology.

[30] Gosmann C., Anahtar M.N., Handley S.A., Farcasanu M., Abu-Ali G., Bowman B.A., Padavattan N., Desai C., Droit L., Moodley A. (2017). Lactobacillus-deficient cervicovaginal bacterial communities are associated with increased HIV acquisition in young South African women. Immunity.

[31] Li J., Zhao F., Wang Y., Chen J., Tao J., Tian G., Wu S., Liu W., Cui Q., Geng B. (2017). Gut microbiota dysbiosis contributes to the development of hypertension. Microbiome.

[32] Peaper D., Bertin J., Eisenbarth S., Gordon J., Flavell R. (2011). NLRP6 inflammasome is a regulator of colonic microbial ecology and risk for colitis. Cell.

[33] Canani R.B., Di Costanzo M., Leone L., Pedata M., Meli R., Calignano A. (2011). Potential beneficial effects of butyrate in intestinal and extraintestinal diseases. World J. Gastroenterol. WJG.

[34] Seth R.K., Kimono D., Alhasson F., Sarkar S., Albadrani M., Lasley S.K., Horner R., Janulewicz P., Nagarkatti M., Nagarkatti P. (2018). Increased butyrate priming in the gut stalls microbiome associated-gastrointestinal inflammation and hepatic metabolic reprogramming in a mouse model of Gulf War Illness. Toxicol. Appl. Pharmacol..

[35] Donohoe D.R., Garge N., Zhang X., Sun W., O’Connell T.M., Bunger M.K., Bultman S.J. (2011). The microbiome and butyrate regulate energy metabolism and autophagy in the mammalian colon. Cell Metab..

[36] Ren Z., Li A., Jiang J., Zhou L., Yu Z., Lu H., Xie H., Chen X., Shao L., Zhang R. (2019). Gut microbiome analysis as a tool towards targeted non-invasive biomarkers for early hepatocellular carcinoma. Gut.

[37] Deng F., McClure M., Rorie R., Wang X., Chai J., Wei X., Lai S., Zhao J. (2019). The vaginal and fecal microbiomes are related to pregnancy status in beef heifers. J. Anim. Sci. Biotechnol..

[38] Loomba R., Seguritan V., Li W., Long T., Klitgord N., Bhatt A., Dulai P.S., Caussy C., Bettencourt R., Highlander S.K. (2017). Gut microbiome-based metagenomic signature for non-invasive detection of advanced fibrosis in human nonalcoholic fatty liver disease. Cell Metab..

[39] Lapidot Y., Amir A., Nosenko R., Uzan-Yulzari A., Veitsman E., Cohen-Ezra O., Davidov Y., Weiss P., Bradichevski T., Segev S. (2020). Alterations in the gut microbiome in the progression of cirrhosis to hepatocellular carcinoma. Msystems.

[40] Lang S., Farowski F., Martin A., Wisplinghoff H., Vehreschild M.J., Krawczyk M., Nowag A., Kretzschmar A., Scholz C., Kasper P. (2020). Prediction of advanced fibrosis in non-alcoholic fatty liver disease using gut microbiota-based approaches compared with simple non-invasive tools. Sci. Rep..

[41] Yang J., McDowell A., Kim E.K., Seo H., Lee W.H., Moon C.-M., Kym S.-M., Lee D.H., Park Y.S., Jee Y.-K. (2019). Development of a colorectal cancer diagnostic model and dietary risk assessment through gut microbiome analysis. Exp. Mol. Med..

[42] Li Q., Chang Y., Zhang K., Chen H., Tao S., Zhang Z. (2020). Implication of the gut microbiome composition of type 2 diabetic patients from northern china. Sci. Rep..

[43] Pecorelli S. (2009). Revised FIGO staging for carcinoma of the vulva, cervix, and endometrium. Int. J. Gynecol. Obstet..

[44] Jung Y., Tagele S.B., Son H., Ibal J.C., Kerfahi D., Yun H., Lee B., Park C.Y., Kim E.S., Kim S.-J. (2020). Modulation of Gut Microbiota in Korean Navy Trainees following a Healthy Lifestyle Change. Microorganisms.

[45] Bolyen E., Rideout J.R., Dillon M.R., Bokulich N.A., Abnet C.C., Al-Ghalith G.A., Alexander H., Alm E.J., Arumugam M., Asnicar F. (2019). Reproducible, interactive, scalable and extensible microbiome data science using QIIME 2. Nat. Biotechnol..

[46] Callahan B.J., McMurdie P.J., Rosen M.J., Han A.W., Johnson A.J.A., Holmes S.P. (2016). DADA2: High-resolution sample inference from Illumina amplicon data. Nat. Methods.

[47] Zakrzewski M., Proietti C., Ellis J.J., Hasan S., Brion M.-J., Berger B., Krause L. (2017). Calypso: A user-friendly web-server for mining and visualizing microbiome–environment interactions. Bioinformatics.

[48] Oksanen J., Blanchet F.G., Kindt R., Legendre P., Minchin P.R., O’hara R., Simpson G.L., Solymos P., Stevens M.H.H., Wagner H. (2013). Package ‘vegan’. Community Ecol. Package Version.

[49] Wickham H. (2007). Reshaping data with the reshape package. J. Stat. Softw..

[50] Wickham H. (2011). ggplot2. Wiley Interdiscip. Rev. Comput. Stat..

[51] Asnicar F., Weingart G., Tickle T.L., Huttenhower C., Segata N. (2015). Compact graphical representation of phylogenetic data and metadata with GraPhlAn. PeerJ.

[52] Smoot M.E., Ono K., Ruscheinski J., Wang P.-L., Ideker T. (2011). Cytoscape 2.8: New features for data integration and network visualization. Bioinformatics.

[53] Wei T., Simko V., Levy M., Xie Y., Jin Y., Zemla J. (2017). Package ‘corrplot’. Statistician.

[54] Peterson B.G., Carl P., Boudt K., Bennett R., Ulrich J., Zivot E., Cornilly D., Hung E., Lestel M., Balkissoon K. (2014). Package ‘PerformanceAnalytics’.

[55] Tibshirani R. (1996). Regression shrinkage and selection via the lasso. J. R. Stat. Soc. Ser. B Methodol..

[56] Wirbel J., Zych K., Essex M., Karcher N., Kartal E., Salazar G., Bork P., Sunagawa S., Zeller G. (2020). SIAMCAT: User-friendly and versatile machine learning workflows for statistically rigorous microbiome analyses. bioRxiv.

[57] RColorBrewer S., Liaw M.A. (2018). Package ‘randomForest’.

[58] Kalyana Chakravarthy S., Jayasudha R., Ranjith K., Dutta A., Pinna N.K., Mande S.S., Sharma S., Garg P., Murthy S.I., Shivaji S. (2018). Alterations in the gut bacterial microbiome in fungal Keratitis patients. PLoS ONE.

[59] Kuhn M. (2008). Building predictive models in R using the caret package. J. Stat. Softw..

[60] Chen H., Boutros P.C. (2011). VennDiagram: A package for the generation of highly-customizable Venn and Euler diagrams in R. BMC Bioinform..

