# Supplementary figures and images for: Dynamics of Fecal Microbiota with and without Invasive Cervical Cancer and Its Application in Early Diagnosis
Source: Cancers (Basel). 2020 Dec 16;12(12):3800. doi: 10.3390/cancers12123800 (PMC7766064; doi:10.3390/cancers12123800)

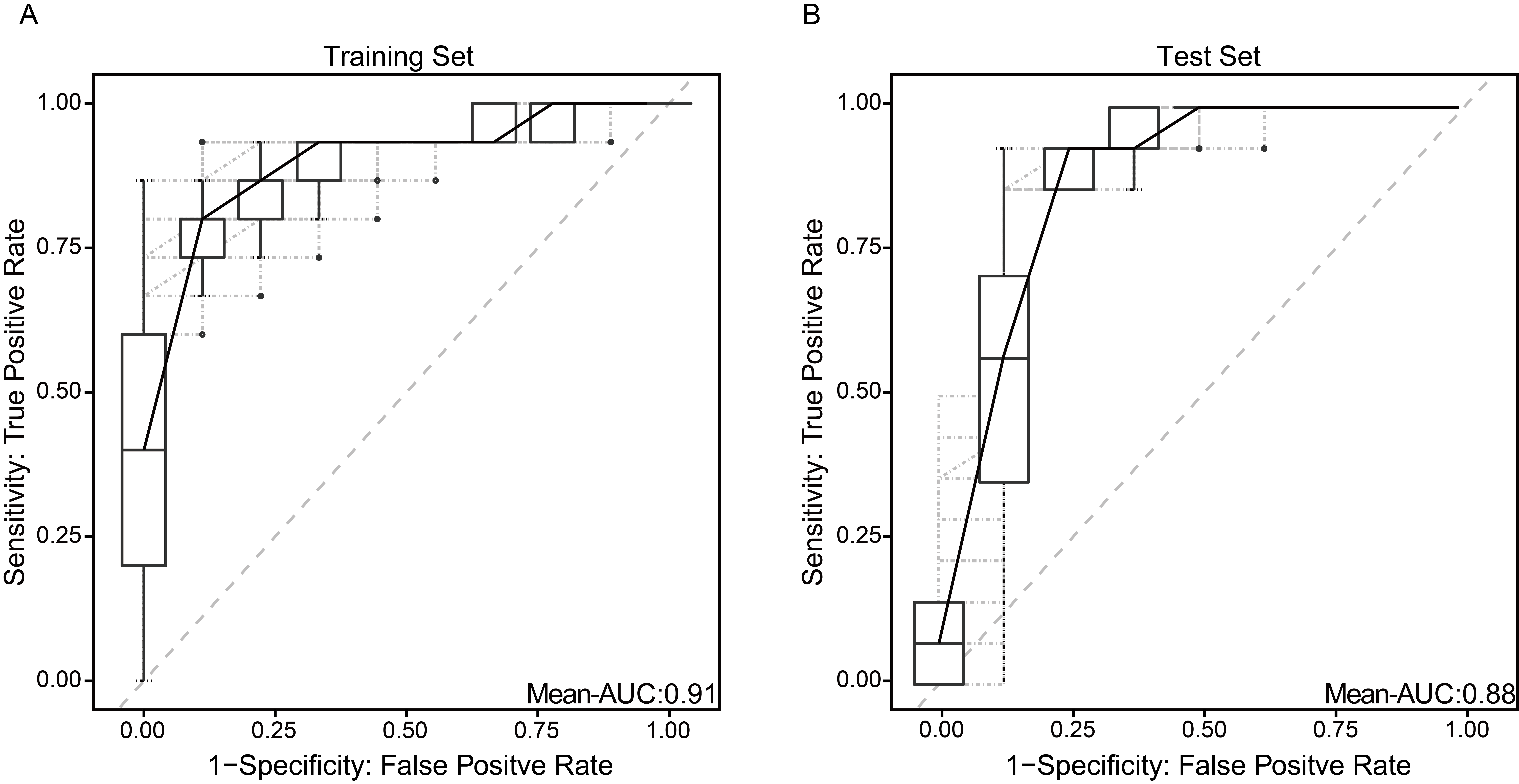

Supplement: Supplementary file 1 [file cancers-12-03800-s001.zip › Figure S4.png]

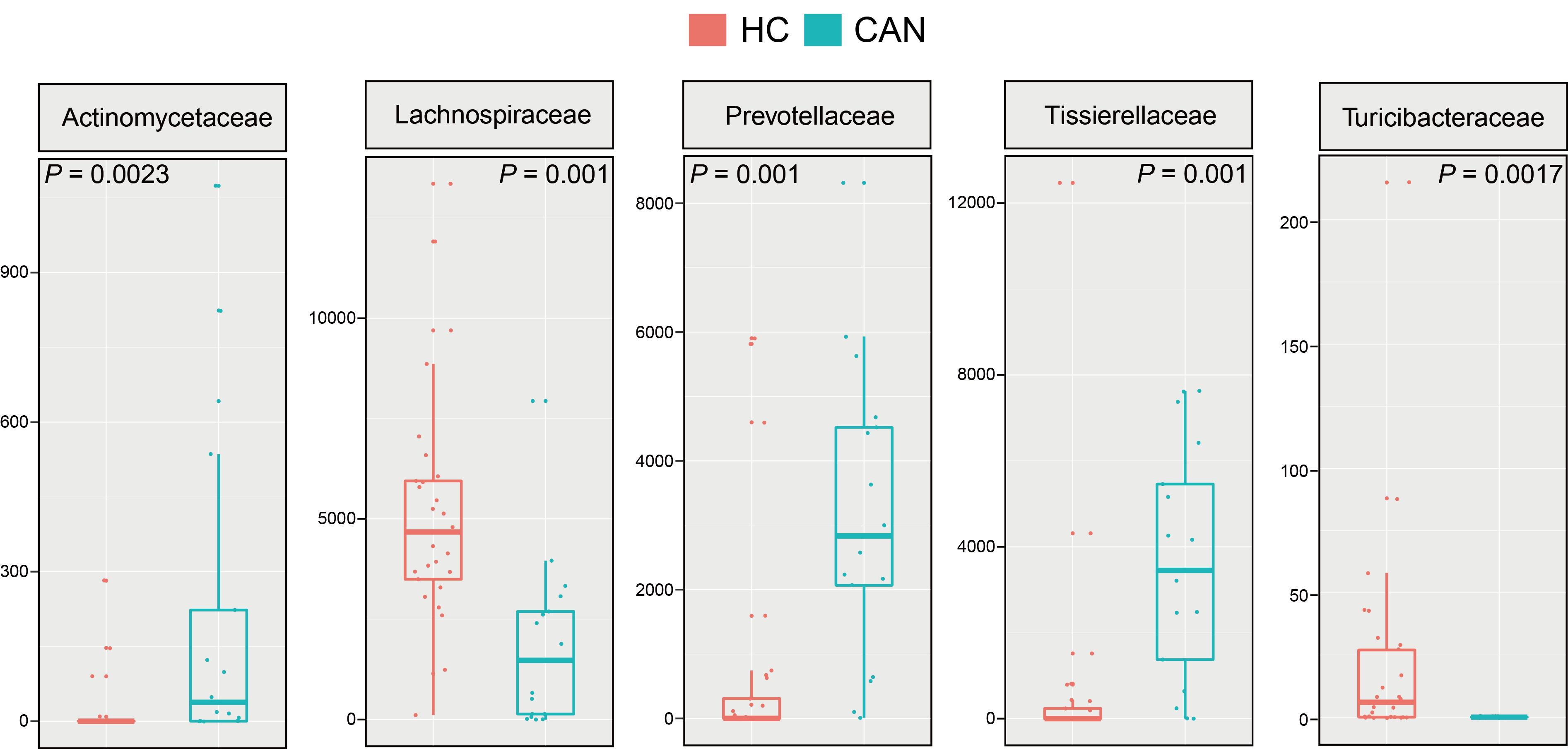

Supplement: Supplementary file 1 [file cancers-12-03800-s001.zip › FIgure S1.png]

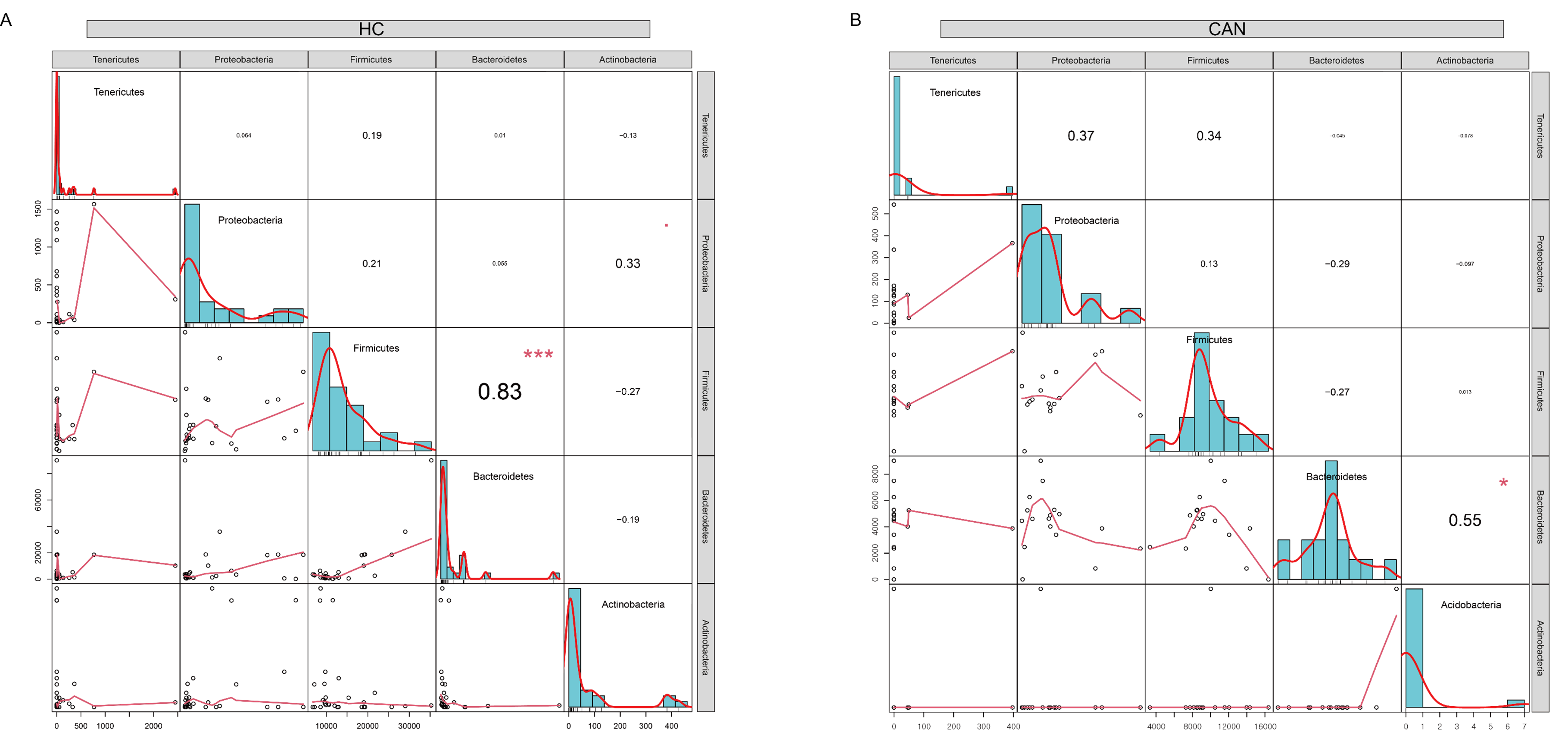

Supplement: Supplementary file 1 [file cancers-12-03800-s001.zip › Figure S2.png]

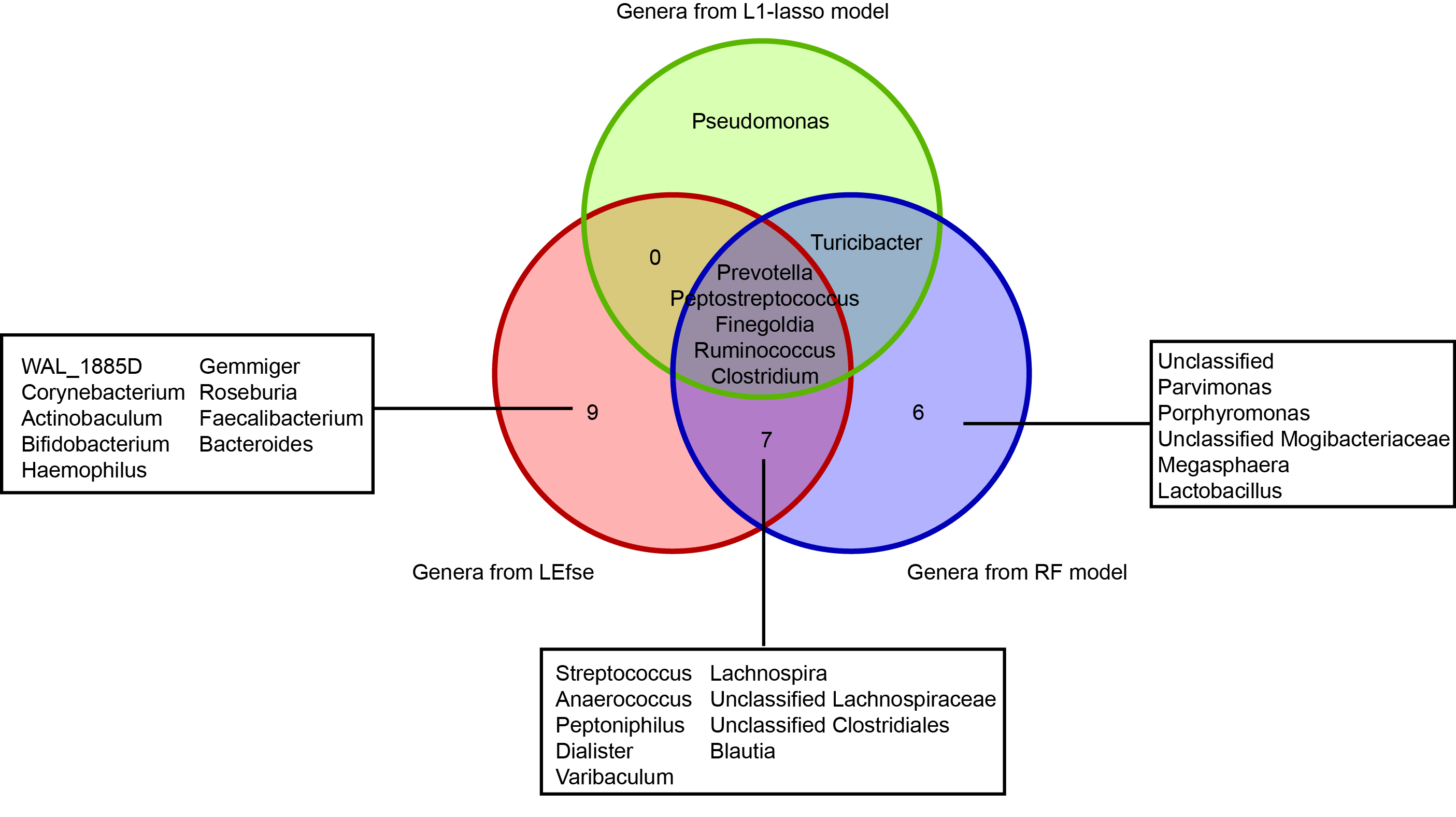

Supplement: Supplementary file 1 [file cancers-12-03800-s001.zip › Figure S3.png]
